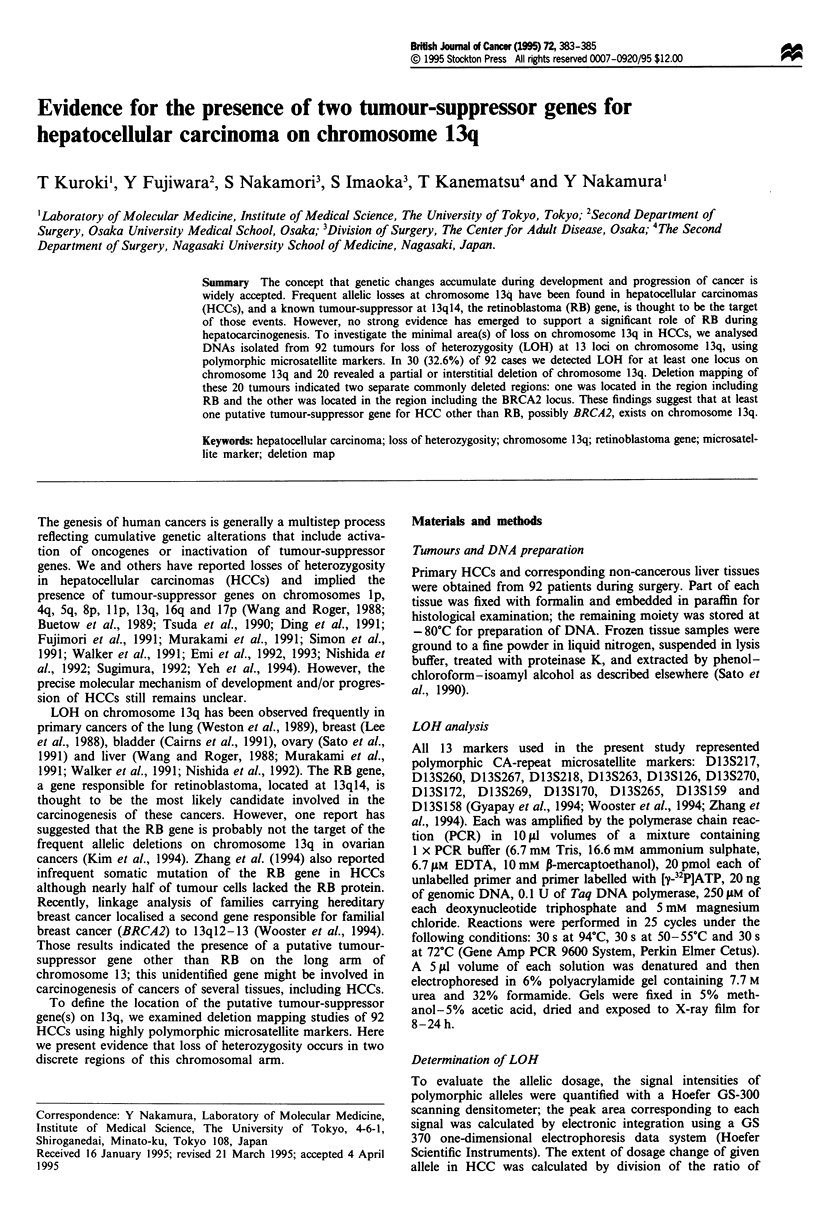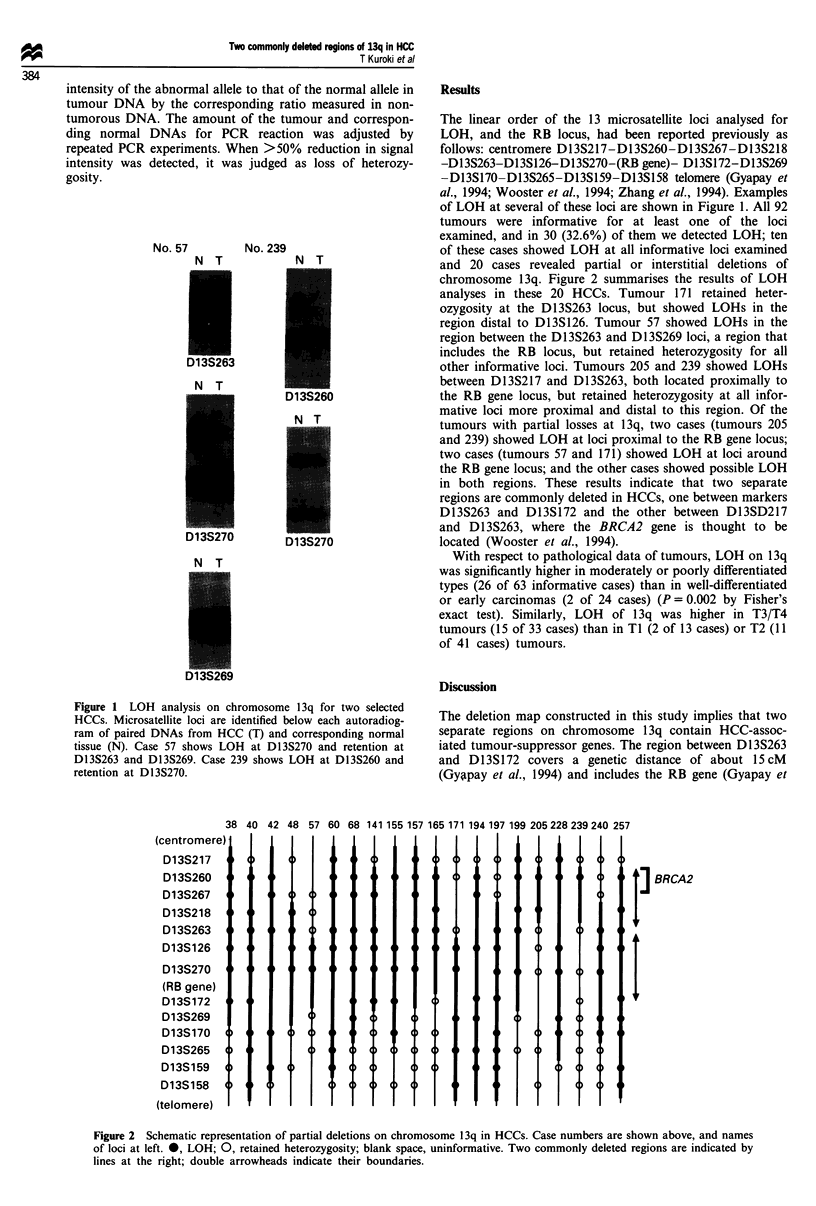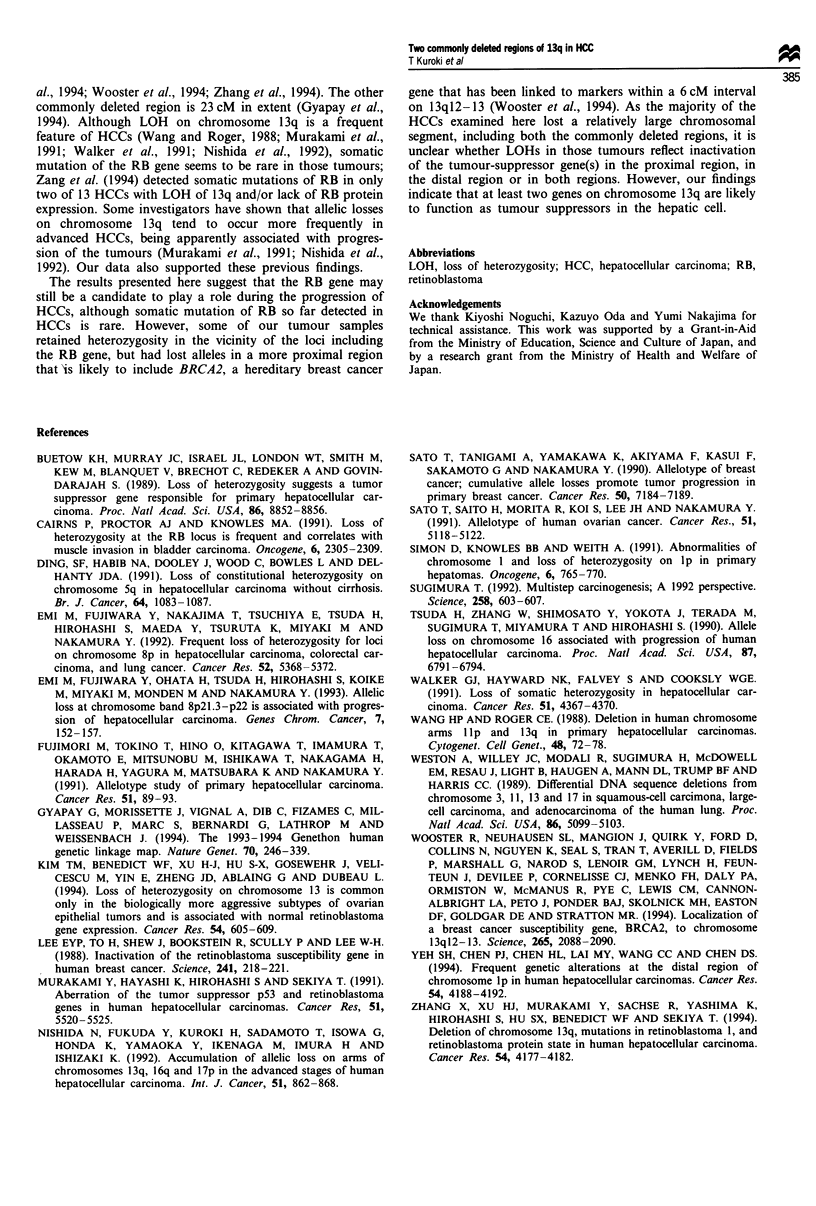# Evidence for the presence of two tumour-suppressor genes for hepatocellular carcinoma on chromosome 13q.

**DOI:** 10.1038/bjc.1995.342

**Published:** 1995-08

**Authors:** T. Kuroki, Y. Fujiwara, S. Nakamori, S. Imaoka, T. Kanematsu, Y. Nakamura

**Affiliations:** Laboratory of Molecular Medicine, University of Tokyo, Japan.

## Abstract

**Images:**


British Journal of Cancer (1995) 72, 383–385
© 1995 Stockton Press All rights reserved 0007–0920/95 $12.00

### Evidence for the presence of two tumour-suppressor genes for hepatocellular carcinoma on chromosome 13q

T Kuroki[1], Y Fujiwara[2], S Nakamori[3], S Imaoka[3], T Kanematsu[4] and Y Nakamura[1]

[1]*Laboratory of Molecular Medicine, Institute of Medical Science, The University of Tokyo, Tokyo;* [2]*Second Department of Surgery, Osaka University Medical School, Osaka;* [3]*Division of Surgery, The Center for Adult Disease, Osaka;* [4]*The Second Department of Surgery, Nagasaki University School of Medicine, Nagasaki, Japan.*

**Summary** The concept that genetic changes accumulate during development and progression of cancer is widely accepted. Frequent allelic losses at chromosome 13q have been found in hepatocellular carcinomas (HCCs), and a known tumour-suppressor at 13q14, the retinoblastoma (RB) gene, is thought to be the target of those events. However, no strong evidence has emerged to support a significant role of RB during hepatocarcinogenesis. To investigate the minimal area(s) of loss on chromosome 13q in HCCs, we analysed DNAs isolated from 92 tumours for loss of heterozygosity (LOH) at 13 loci on chromosome 13q, using polymorphic microsatellite markers. In 30 (32.6%) of 92 cases we detected LOH for at least one locus on chromosome 13q and 20 revealed a partial or interstitial deletion of chromosome 13q. Deletion mapping of these 20 tumours indicated two separate commonly deleted regions: one was located in the region including RB and the other was located in the region including the BRCA2 locus. These findings suggest that at least one putative tumour-suppressor gene for HCC other than RB, possibly *BRCA2*, exists on chromosome 13q.

**Keywords:** hepatocellular carcinoma; loss of heterozygosity; chromosome 13q; retinoblastoma gene; microsatellite marker; deletion map

The genesis of human cancers is generally a multistep process reflecting cumulative genetic alterations that include activation of oncogenes or inactivation of tumour-suppressor genes. We and others have reported losses of heterozygosity in hepatocellular carcinomas (HCCs) and implied the presence of tumour-suppressor genes on chromosomes 1p, 4q, 5q, 8p, 11p, 13q, 16q and 17p (Wang and Roger, 1988; Buetow *et al.*, 1989; Tsuda *et al.*, 1990; Ding *et al.*, 1991; Fujimori *et al.*, 1991; Murakami *et al.*, 1991; Simon *et al.*, 1991; Walker *et al.*, 1991; Emi *et al.*, 1992, 1993; Nishida *et al.*, 1992; Sugimura, 1992; Yeh *et al.*, 1994). However, the precise molecular mechanism of development and/or progression of HCCs still remains unclear.

LOH on chromosome 13q has been observed frequently in primary cancers of the lung (Weston *et al.*, 1989), breast (Lee *et al.*, 1988), bladder (Cairns *et al.*, 1991), ovary (Sato *et al.*, 1991) and liver (Wang and Roger, 1988; Murakami *et al.*, 1991; Walker *et al.*, 1991; Nishida *et al.*, 1992). The RB gene, a gene responsible for retinoblastoma, located at 13q14, is thought to be the most likely candidate involved in the carcinogenesis of these cancers. However, one report has suggested that the RB gene is probably not the target of the frequent allelic deletions on chromosome 13q in ovarian cancers (Kim *et al.*, 1994). Zhang *et al.* (1994) also reported infrequent somatic mutation of the RB gene in HCCs although nearly half of tumour cells lacked the RB protein. Recently, linkage analysis of families carrying hereditary breast cancer localised a second gene responsible for familial breast cancer (*BRCA2*) to 13q12–13 (Wooster *et al.*, 1994). Those results indicated the presence of a putative tumour-suppressor gene other than RB on the long arm of chromosome 13; this unidentified gene might be involved in carcinogenesis of cancers of several tissues, including HCCs.

To define the location of the putative tumour-suppressor gene(s) on 13q, we examined deletion mapping studies of 92 HCCs using highly polymorphic microsatellite markers. Here we present evidence that loss of heterozygosity occurs in two discrete regions of this chromosomal arm.

Correspondence: Y Nakamura, Laboratory of Molecular Medicine, Institute of Medical Science, The University of Tokyo, 4-6-1, Shiroganedai, Minato-ku, Tokyo 108, Japan
Received 16 January 1995; revised 21 March 1995; accepted 4 April 1995

### Materials and methods

### *Tumours and DNA preparation*

Primary HCCs and corresponding non-cancerous liver tissues were obtained from 92 patients during surgery. Part of each tissue was fixed with formalin and embedded in paraffin for histological examination; the remaining moiety was stored at −80°C for preparation of DNA. Frozen tissue samples were ground to a fine powder in liquid nitrogen, suspended in lysis buffer, treated with proteinase K, and extracted by phenol–chloroform–isoamyl alcohol as described elsewhere (Sato *et al.*, 1990).

### *LOH analysis*

All 13 markers used in the present study represented polymorphic CA-repeat microsatellite markers: D13S217, D13S260, D13S267, D13S218, D13S263, D13S126, D13S270, D13S172, D13S269, D13S170, D13S265, D13S159 and D13S158 (Gyapay *et al.*, 1994; Wooster *et al.*, 1994; Zhang *et al.*, 1994). Each was amplified by the polymerase chain reaction (PCR) in 10 μl volumes of a mixture containing 1 × PCR buffer (6.7 mM Tris, 16.6 mM ammonium sulphate, 6.7 μM EDTA, 10 mM β-mercaptoethanol), 20 pmol each of unlabelled primer and primer labelled with [$\gamma$-$^{32}$P]ATP, 20 ng of genomic DNA, 0.1 U of *Taq* DNA polymerase, 250 μM of each deoxynucleotide triphosphate and 5 mM magnesium chloride. Reactions were performed in 25 cycles under the following conditions: 30 s at 94°C, 30 s at 50–55°C and 30 s at 72°C (Gene Amp PCR 9600 System, Perkin Elmer Cetus). A 5 μl volume of each solution was denatured and then electrophoresed in 6% polyacrylamide gel containing 7.7 M urea and 32% formamide. Gels were fixed in 5% methanol–5% acetic acid, dried and exposed to X-ray film for 8–24 h.

### *Determination of LOH*

To evaluate the allelic dosage, the signal intensities of polymorphic alleles were quantified with a Hoefer GS-300 scanning densitometer; the peak area corresponding to each signal was calculated by electronic integration using a GS 370 one-dimensional electrophoresis data system (Hoefer Scientific Instruments). The extent of dosage change of given allele in HCC was calculated by division of the ratio of intensity of the abnormal allele to that of the normal allele in tumour DNA by the corresponding ratio measured in non-tumorous DNA. The amount of the tumour and corresponding normal DNAs for PCR reaction was adjusted by repeated PCR experiments. When >50% reduction in signal intensity was detected, it was judged as loss of heterozygosity.

**Figure 1** LOH analysis on chromosome 13q for two selected HCCs. Microsatellite loci are identified below each autoradiogram of paired DNAs from HCC (T) and corresponding normal tissue (N). Case 57 shows LOH at D13S270 and retention at D13S263 and D13S269. Case 239 shows LOH at D13S260 and retention at D13S270.

### Results

The linear order of the 13 microsatellite loci analysed for LOH, and the RB locus, had been reported previously as follows: centromere D13S217–D13S260–D13S267–D13S218–D13S263–D13S126–D13S270–(RB gene)– D13S172–D13S269–D13S170–D13S265–D13S159–D13S158 telomere (Gyapay *et al.*, 1994; Wooster *et al.*, 1994; Zhang *et al.*, 1994). Examples of LOH at several of these loci are shown in Figure 1. All 92 tumours were informative for at least one of the loci examined, and in 30 (32.6%) of them we detected LOH; ten of these cases showed LOH at all informative loci examined and 20 cases revealed partial or interstitial deletions of chromosome 13q. Figure 2 summarises the results of LOH analyses in these 20 HCCs. Tumour 171 retained heterozygosity at the D13S263 locus, but showed LOHs in the region distal to D13S126. Tumour 57 showed LOHs in the region between the D13S263 and D13S269 loci, a region that includes the RB locus, but retained heterozygosity for all other informative loci. Tumours 205 and 239 showed LOHs between D13S217 and D13S263, both located proximally to the RB gene locus, but retained heterozygosity at all informative loci more proximal and distal to this region. Of the tumours with partial losses at 13q, two cases (tumours 205 and 239) showed LOH at loci proximal to the RB gene locus; two cases (tumours 57 and 171) showed LOH at loci around the RB gene locus; and the other cases showed possible LOH in both regions. These results indicate that two separate regions are commonly deleted in HCCs, one between markers D13S263 and D13S172 and the other between D13SD217 and D13S263, where the *BRCA2* gene is thought to be located (Wooster *et al.*, 1994).

With respect to pathological data of tumours, LOH on 13q was significantly higher in moderately or poorly differentiated types (26 of 63 informative cases) than in well-differentiated or early carcinomas (2 of 24 cases) ($P = 0.002$ by Fisher's exact test). Similarly, LOH of 13q was higher in T3/T4 tumours (15 of 33 cases) than in T1 (2 of 13 cases) or T2 (11 of 41 cases) tumours.

### Discussion

The deletion map constructed in this study implies that two separate regions on chromosome 13q contain HCC-associated tumour-suppressor genes. The region between D13S263 and D13S172 covers a genetic distance of about 15 cM (Gyapay *et al.*, 1994) and includes the RB gene (Gyapay *et*

**Figure 2** Schematic representation of partial deletions on chromosome 13q in HCCs. Case numbers are shown above, and names of loci at left. ●, LOH; O, retained heterozygosity; blank space, uninformative. Two commonly deleted regions are indicated by lines at the right; double arrowheads indicate their boundaries.

al., 1994; Wooster et al., 1994; Zhang et al., 1994). The other commonly deleted region is 23 cM in extent (Gyapay et al., 1994). Although LOH on chromosome 13q is a frequent feature of HCCs (Wang and Roger, 1988; Murakami et al., 1991; Walker et al., 1991; Nishida et al., 1992), somatic mutation of the RB gene seems to be rare in those tumours; Zang et al. (1994) detected somatic mutations of RB in only two of 13 HCCs with LOH of 13q and/or lack of RB protein expression. Some investigators have shown that allelic losses on chromosome 13q tend to occur more frequently in advanced HCCs, being apparently associated with progression of the tumours (Murakami et al., 1991; Nishida et al., 1992). Our data also supported these previous findings.

The results presented here suggest that the RB gene may still be a candidate to play a role during the progression of HCCs, although somatic mutation of RB so far detected in HCCs is rare. However, some of our tumour samples retained heterozygosity in the vicinity of the loci including the RB gene, but had lost alleles in a more proximal region that is likely to include *BRCA2*, a hereditary breast cancer gene that has been linked to markers within a 6 cM interval on 13q12–13 (Wooster et al., 1994). As the majority of the HCCs examined here lost a relatively large chromosomal segment, including both the commonly deleted regions, it is unclear whether LOHs in those tumours reflect inactivation of the tumour-suppressor gene(s) in the proximal region, in the distal region or in both regions. However, our findings indicate that at least two genes on chromosome 13q are likely to function as tumour suppressors in the hepatic cell.

**Abbreviations**

LOH, loss of heterozygosity; HCC, hepatocellular carcinoma; RB, retinoblastoma

**Acknowledgements**

We thank Kiyoshi Noguchi, Kazuyo Oda and Yumi Nakajima for technical assistance. This work was supported by a Grant-in-Aid from the Ministry of Education, Science and Culture of Japan, and by a research grant from the Ministry of Health and Welfare of Japan.

**References**

BUETOW KH, MURRAY JC, ISRAEL JL, LONDON WT, SMITH M, KEW M, BLANQUET V, BRECHOT C, REDEKER A AND GOVINDARAJAH S. (1989). Loss of heterozygosity suggests a tumor suppressor gene responsible for primary hepatocellular carcinoma. *Proc. Natl Acad. Sci. USA*, **86**, 8852–8856.

CAIRNS P, PROCTOR AJ AND KNOWLES MA. (1991). Loss of heterozygosity at the RB locus is frequent and correlates with muscle invasion in bladder carcinoma. *Oncogene*, **6**, 2305–2309.

DING, SF, HABIB NA, DOOLEY J, WOOD C, BOWLES L AND DELHANTY JDA. (1991). Loss of constitutional heterozygosity on chromosome 5q in hepatocellular carcinoma without cirrhosis. *Br. J. Cancer*, **64**, 1083–1087.

EMI M, FUJIWARA Y, NAKAJIMA T, TSUCHIYA E, TSUDA H, HIROHASHI S, MAEDA Y, TSURUTA K, MIYAKI M AND NAKAMURA Y. (1992). Frequent loss of heterozygosity for loci on chromosome 8p in hepatocellular carcinoma, colorectal carcinoma, and lung cancer. *Cancer Res.* **52**, 5368–5372.

EMI M, FUJIWARA Y, OHATA H, TSUDA H, HIROHASHI S, KOIKE M, MIYAKI M, MONDEN M AND NAKAMURA Y. (1993). Allelic loss at chromosome band 8p21.3–p22 is associated with progression of hepatocellular carcinoma. *Genes Chrom. Cancer*, **7**, 152–157.

FUJIMORI M, TOKINO T, HINO O, KITAGAWA T, IMAMURA T, OKAMOTO E, MITSUNOBU M, ISHIKAWA T, NAKAGAMA H, HARADA H, YAGURA M, MATSUBARA K AND NAKAMURA Y. (1991). Allelotype study of primary hepatocellular carcinoma. *Cancer Res.* **51**, 89–93.

GYAPAY G, MORISSETTE J, VIGNAL A, DIB C, FIZAMES C, MILLASSEAU P, MARC S, BERNARDI G, LATHROP M AND WEISSENBACH J. (1994). The 1993–1994 Genethon human genetic linkage map. *Nature Genet.* **70**, 246–339.

KIM TM, BENEDICT WF, XU H-J, HU S-X, GOSEWEHR J, VELICESCU M, YIN E, ZHENG JD, ABLAING G AND DUBEAU L. (1994). Loss of heterozygosity on chromosome 13 is common only in the biologically more aggressive subtypes of ovarian epithelial tumors and is associated with normal retinoblastoma gene expression. *Cancer Res.* **54**, 605–609.

LEE EYP, TO H, SHEW J, BOOKSTEIN R, SCULLY P AND LEE W-H. (1988). Inactivation of the retinoblastoma susceptibility gene in human breast cancer. *Science*, **241**, 218–221.

MURAKAMI Y, HAYASHI K, HIROHASHI S AND SEKIYA T. (1991). Aberration of the tumor suppressor p53 and retinoblastoma genes in human hepatocellular carcinomas. *Cancer Res*, **51**, 5520–5525.

NISHIDA N, FUKUDA Y, KUROKI H, SADAMOTO T, ISOWA G, HONDA K, YAMAOKA Y, IKENAGA M, IMURA H AND ISHIZAKI K. (1992). Accumulation of allelic loss on arms of chromosomes 13q, 16q and 17p in the advanced stages of human hepatocellular carcinoma. *Int. J. Cancer*, **51**, 862–868.

SATO T, TANIGAMI A, YAMAKAWA K, AKIYAMA F, KASUI F, SAKAMOTO G AND NAKAMURA Y. (1990). Allelotype of breast cancer; cumulative allele losses promote tumor progression in primary breast cancer. *Cancer Res.* **50**, 7184–7189.

SATO T, SAITO H, MORITA R, KOI S, LEE JH AND NAKAMURA Y. (1991). Allelotype of human ovarian cancer. *Cancer Res.*, **51**, 5118–5122.

SIMON D, KNOWLES BB AND WEITH A. (1991). Abnormalities of chromosome 1 and loss of heterozygosity on 1p in primary hepatomas. *Oncogene*, **6**, 765–770.

SUGIMURA T. (1992). Multistep carcinogenesis; A 1992 perspective. *Science*, **258**, 603–607.

TSUDA H, ZHANG W, SHIMOSATO Y, YOKOTA J, TERADA M, SUGIMURA T, MIYAMURA T AND HIROHASHI S. (1990). Allele loss on chromosome 16 associated with progression of human hepatocellular carcinoma. *Proc. Natl Acad. Sci. USA*, **87**, 6791–6794.

WALKER GJ, HAYWARD NK, FALVEY S AND COOKSLY WGE. (1991). Loss of somatic heterozygosity in hepatocellular carcinoma. *Cancer Res.* **51**, 4367–4370.

WANG HP AND ROGER CE. (1988). Deletion in human chromosome arms 11p and 13q in primary hepatocellular carcinomas. *Cytogenet. Cell Genet.*, **48**, 72–78.

WESTON A, WILLEY JC, MODALI R, SUGIMURA H, McDOWELL EM, RESAU J, LIGHT B, HAUGEN A, MANN DL, TRUMP BF AND HARRIS CC. (1989). Differential DNA sequence deletions from chromosome 3, 11, 13 and 17 in squamous-cell carcimona, large-cell carcinoma, and adenocarcinoma of the human lung. *Proc. Natl Acad. Sci. USA*, **86**, 5099–5103.

WOOSTER R, NEUHAUSEN SL, MANGION J, QUIRK Y, FORD D, COLLINS N, NGUYEN K, SEAL S, TRAN T, AVERILL D, FIELDS P, MARSHALL G, NAROD S, LENOIR GM, LYNCH H, FEUNTEUN J, DEVILEE P, CORNELISSE CJ, MENKO FH, DALY PA, ORMISTON W, McMANUS R, PYE C, LEWIS CM, CANNON-ALBRIGHT LA, PETO J, PONDER BAJ, SKOLNICK MH, EASTON DF, GOLDGAR DE AND STRATTON MR. (1994). Localization of a breast cancer susceptibility gene, BRCA2, to chromosome 13q12–13. *Science*, **265**, 2088–2090.

YEH SH, CHEN PJ, CHEN HL, LAI MY, WANG CC AND CHEN DS. (1994). Frequent genetic alterations at the distal region of chromosome 1p in human hepatocellular carcinomas. *Cancer Res.* **54**, 4188–4192.

ZHANG X, XU HJ, MURAKAMI Y, SACHSE R, YASHIMA K, HIROHASHI S, HU SX, BENEDICT WF AND SEKIYA T. (1994). Deletion of chromosome 13q, mutations in retinoblastoma 1, and retinoblastoma protein state in human hepatocellular carcinoma. *Cancer Res.* **54**, 4177–4182.